# Brain network mapping and glioma pathophysiology

**DOI:** 10.1093/braincomms/fcad040

**Published:** 2023-02-21

**Authors:** Ayan S Mandal, Steven Brem, John Suckling

**Affiliations:** Perelman School of Medicine, University of Pennsylvania, Philadelphia, PA 19104, USA; Department of Psychiatry, University of Cambridge, Cambridge CB2 0SZ, UK; Perelman School of Medicine, University of Pennsylvania, Philadelphia, PA 19104, USA; Department of Neurosurgery, University of Pennsylvania, Philadelphia, PA 19104, USA; Glioblastoma Translational Center of Excellence, Abramson Cancer Center, Philadelphia, PA 19104, USA; Department of Psychiatry, University of Cambridge, Cambridge CB2 0SZ, UK

**Keywords:** glioma, network neuroscience, brain mapping, neuro-oncology, connectivity

## Abstract

Adult diffuse gliomas are among the most difficult brain disorders to treat in part due to a lack of clarity regarding the anatomical origins and mechanisms of migration of the tumours. While the importance of studying networks of glioma spread has been recognized for at least 80 years, the ability to carry out such investigations in humans has emerged only recently. Here, we comprehensively review the fields of brain network mapping and glioma biology to provide a primer for investigators interested in merging these areas of inquiry for the purposes of translational research. Specifically, we trace the historical development of ideas in both brain network mapping and glioma biology, highlighting studies that explore clinical applications of network neuroscience, cells-of-origin of diffuse glioma and glioma–neuronal interactions. We discuss recent research that has merged neuro-oncology and network neuroscience, finding that the spatial distribution patterns of gliomas follow intrinsic functional and structural brain networks. Ultimately, we call for more contributions from network neuroimaging to realize the translational potential of cancer neuroscience.

## Teaser

Mandal *et al.* review the burgeoning field of cancer neuroscience as studied using human brain mapping. They outline key historical developments in functional neuroimaging and glioma biology, respectively, highlighting several areas where the two fields can continue to be merged.

## Graphical Abstract

**Graphical Abstract:**
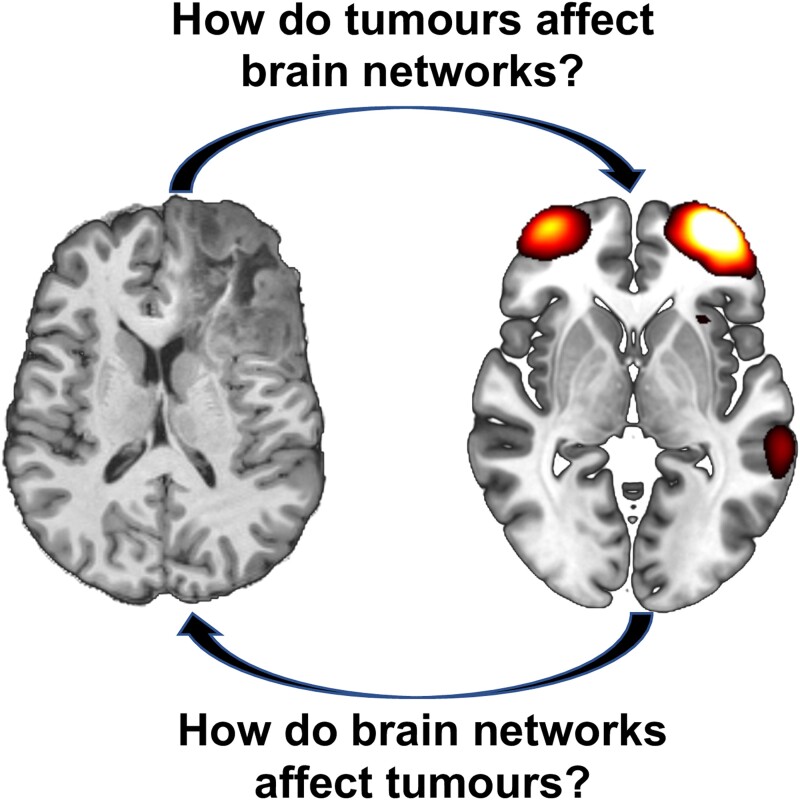

## Introduction

‘The great practical significance of an exact knowledge of the forms of growth and extension of gliomas needs no special emphasis. For both the neurologist and the neurosurgeon this knowledge is certainly one of the most important aspects of the pathology of the gliomas. It is therefore surprising that this aspect of the problem has been rather neglected in modern research on this subject.’—Hans Scherer, *Brain*, 1940^1^

For at least 80 years, the study of glioma origins and migration has been a topic of clinical import yet limited scientific fruit. Even today, it could be argued that this aspect of the problem has not received commensurate attention given the potential implications of a complete understanding of glioma migration patterns for clinical treatment of primary brain tumours. One reason why a systems-level understanding of glioma development has yet to be achieved is because the technologies necessary to study brain disease from a top-down approach have emerged only recently. In other areas of neurology, network-based models of disease spread and cognitive impact were proposed well before the technologies to rigorously test the relevant hypotheses existed.^2–5^ In a similar way, the Belgian neuropathologist, Hans Scherer, could not dream of providing *in vivo* evidence for his proposals of glioma migration along brain networks, presenting only static pathology slides to support his claims. As the ability to non-invasively map brain networks in humans reaches maturity, it is well worth revisiting the literature of modern neuroscience's predecessors to test their prescient ideas.

In this review, we therefore aim to introduce the reader to two emerging fields—human brain mapping and glioma biology—that can be synthesized to provide insights into the origins and development of glioma tumours. Many techniques exist in human brain mapping, including structural, diffusion and functional MRI (fMRI) as well as electroencephalography (EEG), magnetoencephalography (MEG) and electrocorticography. Each of these techniques has produced relevant and important data regarding glioma pathophysiology. The prior articles (Table 1) review the clinical applications of brain imaging with these modalities to study glioma. In this review, we focus on the background and application of functional and structural brain network mapping to investigate glioma pathophysiology. The ultimate goal of this work is to provide a foundation within the burgeoning field of cancer neuroscience^13^ for the application of non-invasive imaging of brain structure and function in the scientific study and clinical management of diffuse gliomas.

**Table 1:** Prior reviews on advanced neuroimaging of gliomas relevant to surgical planning, connectomics and functional/electrophysiological monitoring

Article	Description	Modalities reviewed
Aerts *et al.*, 2016^6^	A review of the impact of brain lesions (e.g. tumours, infarcts, and traumatic brain injuries) on the connectome. While lesions significantly alter topological characteristics of brain networks (defined using DWI, fMRI or MEG data), such effects depend critically on lesion location.	DWI, fMRI, MEG, EEG
Henderson *et al.*, 2020^7^	An overview of the state of the art in tractography for surgical planning of brain tumour resections. Covers critical tools and approaches to enhance crossing fibre visualization, correct for peritumoural oedema and automatically identify tracts.	DWI
Verburg & de Witt Hammer, 2020^8^	An overview of imaging techniques used to plan brain tumour resections so as to maximize extent of resection while preserving brain function.	DWI, fMRI, MEG, EEG, MRS
Sanvito *et al.*, 2021^9^	An introduction to the use of brain imaging tools to unravel molecular features relevant to glioma pathogenesis, such as composition of the tumour tissue microenvironment or biomarker monitoring (e.g. 2-hydroxyglutarate spectroscopy).	DWI, PWI, MRS
Osorio & Aghi, 2014^9^	A review of different preoperative and intraoperative imaging modalities useful for planning surgical resection of gliomas.	DWI, fMRI, MEG
Hart *et al.*, 2015^10^	An introduction to the application of graph theory connectome analysis in neurosurgery.	DWI, fMRI, MEG, EEG
Aabedi *et al.*, 2022 ^11^	A review of preoperative and intraoperative techniques useful for handling the involvement of white matter language tracts in glioma. Also covers the role of white matter plasticity in recovery from injury and glioma pathophysiology.	DWI, fMRI, MEG
Azad & Duffau, 2020^12^	Highlights the limitations of preoperative DWI and fMRI imaging in patient selection and surgical planning. Covers the low sensitivity and specificity of fMRI compared to DES, as well as the notion that as a structural imaging modality, DWI cannot provide functional interrogation of white matter pathways.	DWI, fMRI

## Brain mapping: introduction

### Resting-state fMRI

**Figure 1:**
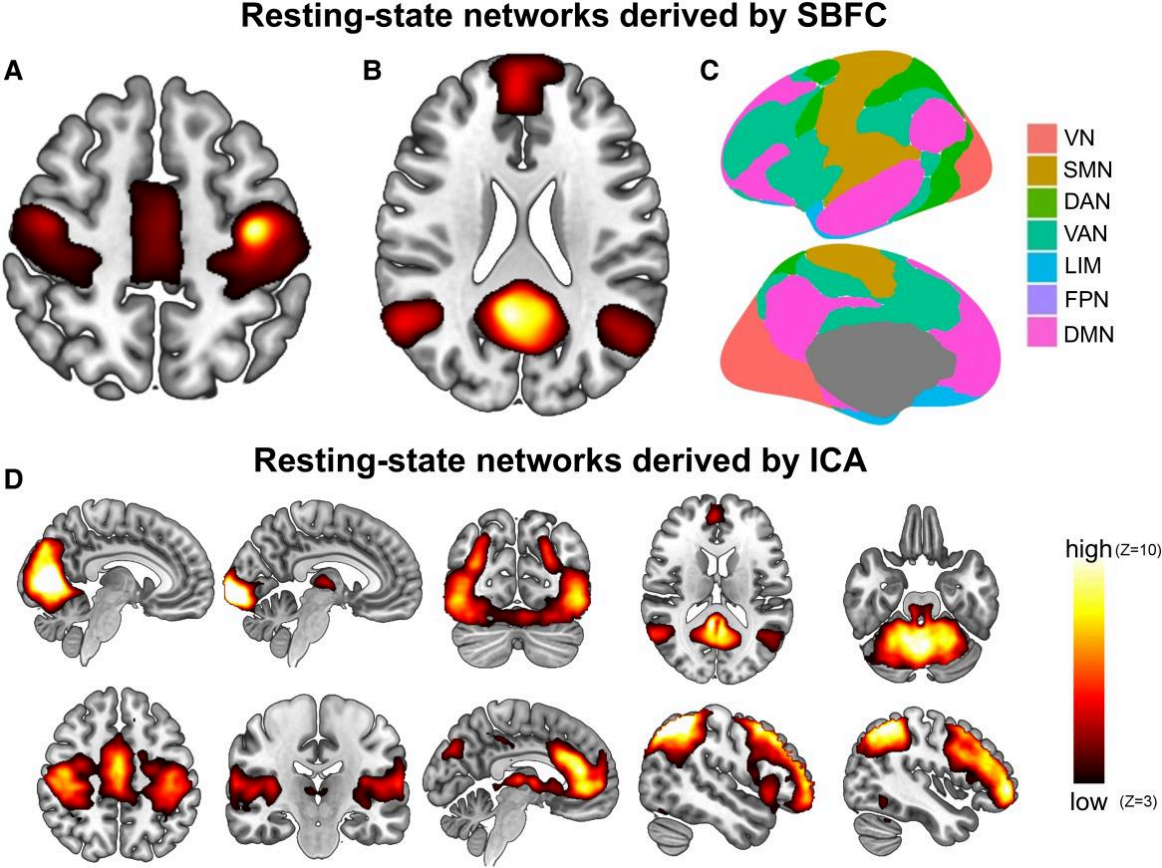
**Intrinsic brain networks derived from resting-state functional connectivity.** (**A**) Sensorimotor network derived from seed-based functional connectivity of a motor cortex voxel using Neurosynth.^16^ (**B**) Default mode network derived from seed-based functional connectivity of a posterior cingulate cortex voxel using Neurosynth. (**C**) Canonical resting-state networks from Yeo and colleagues^27^ derived from clustering of seed-based functional connectivity maps averaged across 1000 college-aged individuals. Figure created using ggseg (https://github.com/ggseg/ggseg). (**D**) Resting-state networks derived from ICA of fMRI data across 36 subjects, which were shown to be concordant with task-based activations from thousands of fMRI experiments involving over 30 000 subjects.^17^ SBFC, seed-based functional connectivity; ICA, independent component analysis; VN, visual network; SMN, sensorimotor network; DAN, dorsal attention network; VAN, ventral attention network; LIM, limbic network; FPN, frontoparietal network; DMN, default mode network.

Non-invasive mapping of human brain networks was first described in 1995 with Biswal and colleagues’^14^ reports of temporally correlated, blood–oxygen level dependent (BOLD) signals within the motor cortex. At this point in history, the BOLD signal was widely interpreted within the context of task-induced elevations, topographically organized upon brain tissue relevant for performing a given task; it was not yet clear how to interpret covariance in this signal across brain regions during the resting state (i.e. in the absence of a task). At first, the nature of these correlations was suspected to be artefactual by many of Biswal's colleagues^15^, with one senior investigator complaining that his confusing results were an ill consequence of allowing graduate students to design experiments. However, through subsequent, carefully designed studies, Biswal ruled out confounding vascular, head motion and respiratory signals as primary drivers of these spatiotemporal BOLD correlations, and the community grew increasingly more accepting of the view that these patterns could reflect neuronal connectivity. The pattern of BOLD correlations identified by Biswal came to be known as the ‘sensorimotor network’ and is displayed in Fig. 1A.

The discovery of the default mode network (DMN) represents the next major milestone in the conceptual development of resting-state functional connectivity. The DMN had first been described as a ‘task-negative’ network of association cortical regions that appeared deactivated in experimental functional imaging studies whenever a task condition was contrasted with the resting state.^18^ However, instead of interpreting this constellation of regions as ‘deactivated’ during attention-demanding cognitive tasks, Raichle and colleagues^19^ proposed that they could instead represent a network of regions that were ‘activated’ in the resting state, reflecting an introspective, ‘default’ mode. A few years later, Michael Greicius and colleagues^20^ produced the first map of the DMN derived from a resting-state functional connectivity analysis by applying the seed-based approach introduced by Biswal to identify the network of brain regions functionally correlated with the posterior cingulate cortex (Fig. 1B). Currently, the identification of the DMN represents one of the most robust findings in neuroscience, readily apparent from relatively simple analyses of resting-state functional imaging data from almost any participant, regardless of their neurological status.

Delineation of the DMN soon led to the exploration of other large-scale functional connectivity networks using two complementary approaches. The first approach, which has already been described, is called seed-based, functional connectivity; this involves correlating the BOLD signal of each grey matter voxel with a reference waveform, or seed, which could be from a single voxel or an averaged time-series over a collection of voxels. Seeds from adjacent brain regions can produce distinct maps of functional connectivity, which makes this approach useful for unveiling the diversity of brain functional networks (Fig. 1C).^21^ However, the tight dependence on seed location is also a limitation of the seed-based approach, since slight differences in the registration of a seed between subjects can result in the identification of distinct networks.^22^ A second approach utilizes a source-separation, dimensionality reduction technique called independent component analysis (ICA) to decompose fMRI data into components of signal and noise.^23–25^ ICA is used both to denoise data and identify resting-state functional networks, which naturally manifest as the signal components from the analysis (Fig. 1D). ICA requires few *a priori* assumptions compared to the seed-based approach, yet one important algorithmic choice is the intended number of resulting components, which can be handled a number of different ways.^23,26^

Several studies have applied seed-based approaches to comprehensively map the human brain's resting-state networks. Perhaps the most impactful of these approaches was carried out by Thomas Yeo and colleagues^27^ in 2011. Using resting-state fMRI (rs-fMRI) data from 1000 college-aged individuals, as well as sophisticated surface-based registration techniques, Yeo *et al.* identified two optimal clustering solutions which decomposed the cerebral cortex into a fixed set of parsimonious networks: a 7-network solution and a 17-network solution. Most subsequent investigators have focused on the seven-network solution for its simplicity and useful insights into brain organization (Fig. 1C). The canonical, Yeo networks comprise of two networks which cover primary cortical regions, namely the visual and somatosensory cortices, as well as five that span broad swaths of association cortex, including the dorsal attention, ventral attention, limbic, frontoparietal and DMN. Other attempts at mapping the resting-state networks from the seed-based approach have identified similar network decompositions. These approaches have been used to map functional brain networks in glioma patients preoperatively, hinting at the potential translation of this technique to guide surgical planning.^7,28,29^

### Historical development of diffusion tractography

Diffusion tractography is a method to visualize white matter tracts in the brain from diffusion MRI sequences.^30^ The technique relies on the fact that water diffuses anisotropically (i.e. in one direction) through white matter tracts because of the hydrophobic composition of myelin sheaths. Thus, diffusion MRI can provide insight into the locations of large-scale white matter pathways by tracking the movement of water molecules.

Diffusion tensor imaging was developed by Basser *et al.*^31^ in 1994 and reflects one of the first attempts to visualize white matter pathways using diffusion MRI data. The application of the diffusion tensor allows the researcher to model the shape (i.e. direction) of water diffusion in such a way that can track the complex geometry of white matter pathways in the brain.^30^ A diffusion probability density function can be computed for each voxel, which is exploited to trace the trajectory of a white matter tract.^32^

**Figure 2:**
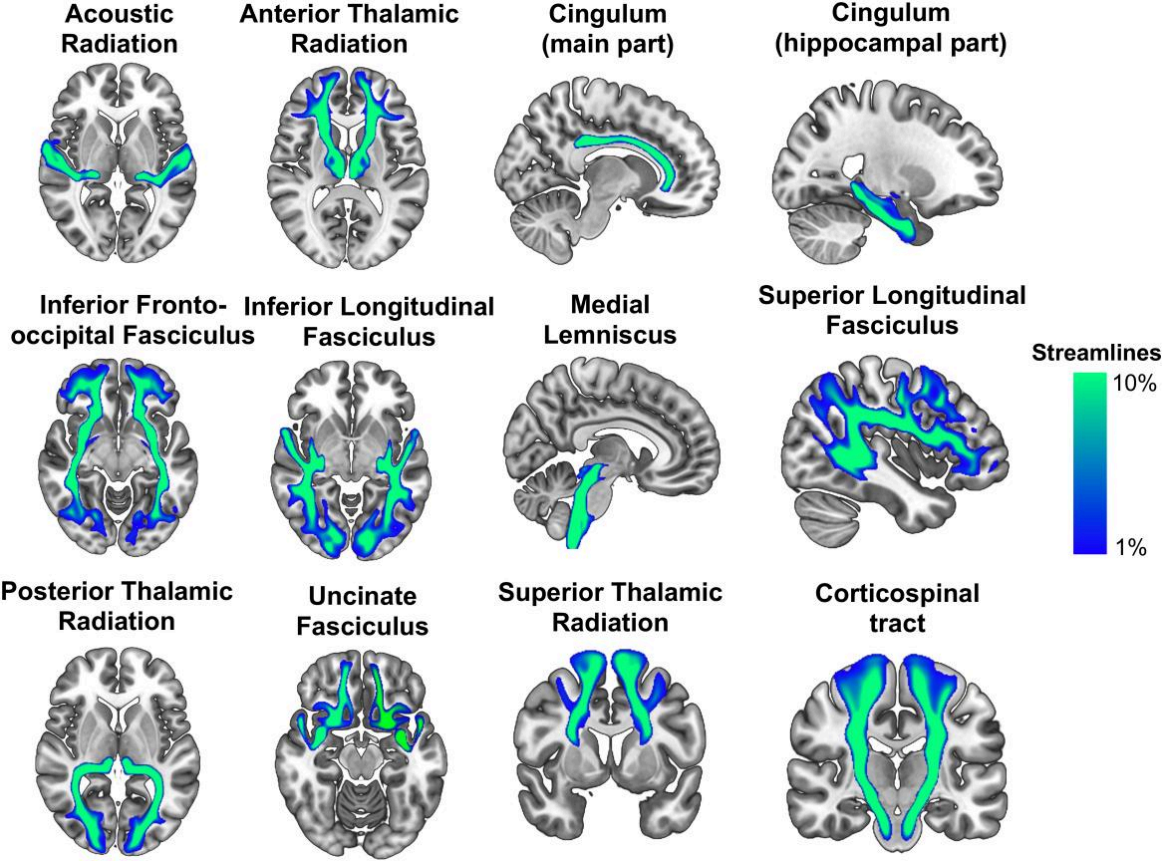
**Structural brain networks derived from diffusion tractography.** Bilateral white matter pathways mapped from UK Biobank diffusion imaging data run through the XTRACT standardized protocols.^34^ Brain map values reported as a percentage of max number of streamlines.

Two approaches to diffusion tractography exist: ‘deterministic’ and ‘probabilistic’ tractography. Deterministic tractography attempts aims to delineate a white matter tract by starting at a seed point, then following a single direction at each voxel to track the pathway throughout the brain.^32^ Probabilistic tractography samples a large number of different possible pathways from a starting seed point based on the diffusion probability density function at each voxel.^33^ Each approach has its own advantages and disadvantages. Probabilistic tractography is more resource-intensive given the large number of simulated pathways, but it also more comprehensively covers each possible structural connection to a given seed location.^32^ An array of canonical white matter pathways that can be mapped by probabilistic tractography are shown in Fig. 2.^34^

Diffusion tensor imaging was improved upon in the 2000s with the development of techniques like diffusion spectrum imaging and high angular resolution diffusion imaging, which address the fact that multiple white matter fibres often cross at a single voxel.^35,36^ Although prior studies have highlighted a suboptimal false positive rate in diffusion tractography, advances in image acquisition like multishell diffusion imaging allow for more accurate results.^37^ It is also worth noting that different research teams can derive slightly different white matter bundles from the same diffusion-weighted imaging dataset, simply because different groups have different protocols and processing pipelines.^38^ Some groups have published high-quality, standardized workflows with openly available software to address this problem.^34^ The results of diffusion tractography can also be compared to human neuroanatomical atlases derived from post-mortem brain tissue to address any ambiguity.^39^ Overall, diffusion tractography reflects an extremely useful technique for mapping *in vivo* structural connectivity, with numerous potential clinical applications, including in the resection of gliomas (which often live in the white matter).

In 2005, the term ‘connectome’ was coined simultaneously by Hagmann^40^ and Sporns *et al.*^41^ to describe the full collection of the brain's connections. While first applied to maps of structural connectivity, soon this term was also invoked to describe the catalogue of functional connections present in any given individual's brain as elucidated by resting-state functional imaging. Although it was well-recognized that interindividual variation should exist across connectomes, a tantalizing goal of cognitive neuroscience arose to uncover the general wiring diagram of the human brain. In 2009, the National Institute of Health funded two consortiums primarily aimed at understanding functional and anatomical connectivity, respectively, in a large-scale effort called the Human Connectome Project.^42^ Many positive outcomes came from this project including improved image acquisition, processing and visualization techniques,^43,44^ a multimodal brain parcellation^45^ as well as a detailed, ‘wiring diagram’ of the healthy brain in early adulthood.^46^ A similarly ambitious imaging study was initiated by the UK Biobank in 2014 which mapped the healthy connectome in late adulthood.^47,48^ The quantity and quality of data describing ‘normal’ brain organization, characterized throughout the lifespan, now provide the unprecedented opportunity to better understand how the brain diverges in disease, with implications for the clinical management of brain tumours.

### Graph theoretical applications in network neuroscience

Brain network analysis has several potential applications in neuro-oncology, from guiding tumour resections^49^ to unpacking intercellular dynamics that influence glioma growth.^50^ To understand brain networks, it is useful to consult other areas of mathematics and science that have developed a toolset and vocabulary to characterize networks. The branch of mathematics concerned with networks is called graph theory.^51,52^ Graph theory is the study of graphs, which are mathematical objects composed of nodes (also called vertices) and edges (also called links) that each connect two nodes. A graph can be thought of as the mathematical representation of a real-world network. Conceiving of networks in the abstract, as graphs, has been useful in the fields of computer science, molecular biology, physics and sociology,^53–60^ prompting the application of this approach to systems neuroscience.^61,62^ When graph theory is used to interpret functional networks derived from resting-state imaging, the nodes of the graph typically represent broad brain regions as defined by a chosen parcellation scheme, while the edges reflect some measure of the statistical relationship (e.g. Pearson's correlation, mutual information, etc.) of the BOLD signals at two nodes.^62,63^ These edges can be either weighted by the strength of the relationship or binarized by some threshold. Since the directionality of the relationship is difficult to assess in fMRI due to the limited temporal resolution, the edges in graphs based on rs-fMRI data tend to be undirected, although in network science more broadly edges can have directionality. Negatively weighted edges remain enigmatic and are often ignored. A directly analogous approach can be applied to EEG, MEG or electrocorticography, where signal power within frequency bands that relate to neural firing can be correlated across distinct nodes.^64^

Within the context of brain networks, there are three general properties of interest: integration, segregation and centrality.^63^ Network statistics that assess each property are described in Table 2. ‘Integration’ refers to the efficiency of a network, where efficiency is generally related to the average distances between nodes of the graph; therefore, a well-integrated or efficient network is one where travelling between any two given nodes is a relatively short journey. ‘Segregation’ generally refers to the extent to which a network is organized within units of strong intraconnectivity. Measures of segregation vary in how large these units are assumed to be, with metrics like clustering coefficient tracking the number of triangles (smallest possible fully connected unit) in a given network,^65^ while metrics like modularity assess the intraconnectivity of larger clusters of nodes compared to the connectivity between nodes in different modules.^60,66^

**Table 2:** Descriptions of graph theory measures

Term	Description	Global or local?
**Measures of integration**		
Global efficiency	Average inverse shortest path length. Path length is operationalized as the inverse of the weights in a weighted network, A high global efficiency denotes a well-integrated network.	Global
Characteristic path length	Average shortest path length. A low characteristic path length denotes a well-integrated network.	Global
**Measures of segregation**		
Clustering coefficient	Fraction of a node's neighbours that are also neighbours. Can be averaged across all nodes in the network to provide a global measure of segregation.	Local
Modularity	Degree to which a network can be subdivided into groups of nodes (i.e. modules) with maximal intraconnectivity and minimal inter-connectivity. Cannot be calculated directly, but rather after optimal modular structure has been determined by an optimization algorithm.	Global
**Measures of centrality**		
Degree	Number of edges involving a given node. Applied to binary graphs. A node with high degree can be interpreted as a hub.	Local
Strength	Sum of weighted edges involving a given node. Applied to weighted graphs. A node with high strength can be interpreted as a hub.	Local
Betweenness centrality	Fraction of all shortest paths to pass through a given node. A node with a high betweenness centrality can be interpreted as a hub.	Local
Participation coefficient	Distribution of a node's edges with other nodes in different modules versus the same module. A high participation coefficient denotes a ‘connector hub’, which help link distinct modules.	Local
Within module Z-score	*Z*-score of nodal strength within a given node's module. A high within-module *Z*-score denotes a ‘provincial hub’, important for integration within a given module.	Local

Local network statistics describe properties of individual nodes, whereas global statistics describe properties of whole networks.

Finally, ‘centrality’ refers broadly to the importance of a given node to the network, where ‘importance’ can be defined in several ways. The most straightforward measure of centrality is ‘nodal degree’ which the total number of connections a node makes with other nodes. A related measure is ‘nodal strength’, which applies to weighted graphs and refers to the sum of weights involving a given node. Another important metric of centrality is ‘betweenness centrality’, which refers to the fraction of all shortest paths that involve a given node.^67^ This measure is closely related to ‘local efficiency’, which quantifies the importance of a node to the network's global efficiency.^68^ Finally, the centrality of a node can be assessed by their intermodular connectivity, through metrics like ‘participation coefficient’.^66^ Nodes that help connect different modules together are called ‘connectors’, whereas nodes that are highly connected within their own module are described as ‘provincial’.^69^ All three classes of centrality measures described above are used to define hubs, or especially well-connected nodes, of a network, which are of broad importance in both healthy brain function and pathological dysfunction.

#### Characteristics of brain networks

Among the first developments in network neuroscience was the discovery of small world brain networks.^62,65^ Small-worldness is defined as some ratio between the integration and segregation of a network. A network with an optimal balance of both is one where each node has a relatively short path to every other node, while organization into clusters or modules is still maintained.^65,70^ Based on simulations comparing empirical brain networks to surrogate networks with preserved distributional properties yet randomized topology, networks describing nervous systems across many species, ranging from the *Caenorhabditis elegans* neuronal connectome to the human resting-state functional connectome, have been observed to possess significant small-worldness.^71^

What does small-worldness mean for brain organization? In spatially embedded networks, such as the brain, high levels of integration are essentially always expected. Nodes that are close to one another in space tend to be connected, given the low physiological cost of forming connections with close-by nodes, resulting in high levels of clustering. More interestingly, brain networks tend to also possess long-distance connections, which often involve multiple modules.^72^ These long-distance connections are responsible for the small-worldness of brain networks, as they allow for efficient communication between distant brain regions.^62^ To support long-distance communication, small-world networks will typically also possess hubs, which mediate the paths between nodes in their local neighbourhood and far-away modules.^69,73^

While hubs support efficient information transfer in spatially embedded networks, they come at a cost.^74^ By definition, hubs form many connections, which require constant grooming throughout development dedicated to axon myelination and synaptogenesis. In addition, hubs also form long-distance connections, which are especially physiologically expensive to maintain, since proteins in the presynaptic terminal are manufactured in the cell body, and thus must travel the full distance of the axon to reach their target. Considering this energy constraint, some authors proposed that brain networks have been sculpted over the course of evolution to meet an optimal balance between maximizing topological efficiency and minimizing wiring costs.^75^ Hubs are essential for maintaining this trade-off, yet because of their disproportionate metabolic demand, these regions are vulnerable to neurological and psychiatric disease (as discussed below in the ‘Clinical applications’ section).

A major question of interest is how functional and structural brain network topology relate to one another. Functional connections are facilitated by an underlying structural network that mediates communication between distant neuronal populations.^76^ As such, structural and functional networks are reasonably well-correlated, with both types of networks bearing similar characteristic features like small-worldness and hub organization.^77,78^ Nevertheless, there are important differences between structural and functional networks since functional connectivity is more dynamic and captures higher-order interactions mediated by multisynaptic connections.^78^ Many pathologies affect both structural and functional connectivity, so it is important to bear in mind that the two are closely interrelated.

### Clinical applications

Resting-state functional connectivity studies have furthered our basic understanding of human brain function, advancing a more nuanced perspective compared to the localizationist view that preceded this approach. How has this technique improved our understanding of brain disorders? Translational research in network neuroscience can be understood as comprising two main arms targeted at clarifying the pathophysiology of neuropsychiatric disease as well as improving treatment planning for surgical and stimulation interventions.

#### Pathophysiology

Despite the prominence of focal lesion studies throughout the history of neurology, the notion that brain diseases cause symptoms by affecting large-scale networks is quite old. Both Wernicke^2^ and Geschwind^3^ composed brain network models to explain the cognitive deficits associated with neuropsychiatric disorders such as aphasia and schizophrenia. Current techniques to map brain networks *in vivo* provide the first opportunity to experimentally examine the relationship between connectivity and disease.

One of the first seminal studies in ‘pathoconnectomics’ linked the spatial patterning of neurodegeneration in Alzheimer's disease to the DMN. Buckner and colleagues^79^ used PET imaging to localize amyloid beta, one of the main pathogenic proteins in Alzheimer's disease, and found that the biomarker co-localized with nodes of the DMN, most prominently the posterior cingulate cortex. This work was extended by Seeley and colleagues^80^ in 2009, who demonstrated that brain atrophy in a wide range of neurodegenerative conditions localized to large-scale brain networks.

Finally, a seminal study from Crossley and colleagues^81^ demonstrated that the link between disease and connectivity extends even further. Using a large database summarizing the results from 392 of published voxel-based morphometry studies, they found that the brain regions which tend to be atrophied in a litany of neurological disorders co-localize with the hubs of the structural connectome. Although many conditions implicate divergent regions of the brain (e.g. Alzheimer's disease and schizophrenia), each condition still tends to affect regions around a brain hub. Crossley and colleagues also reported that this phenomenon generalized to functional network hubs, as the functional connectome shared many of the same topological properties of their structural connectome.

What insights into brain pathology do these findings provide? Two non-mutually exclusive hypotheses have been proposed to explain the link between brain disease and connectivity. First, as discussed previously, brain network hubs require a disproportionate amount of energy to maintain, which could leave these areas vulnerable to disease.^61,75^ Intense metabolic demand has been proposed to contribute to the vulnerability of upper motor neurons to degeneration in amyotrophic lateral sclerosis.^82^ In a similar way, the propensity for neurological diseases to implicate hubs could be a consequence of the economy of brain network organization, in which the hubs are left vulnerable to metabolic disruption.^75^

A second possibility, which may only apply to a subset of neurological diseases, is that the pathology could spread along brain networks. Prion-like transneuronal degeneration has been demonstrated in animal models of neurodegenerative conditions like frontotemporal dementia, Parkinson's disease and Alzheimer's disease.^83–87^ Some groups have shown, both in animal models and in patients, that longitudinal brain atrophy can be predicted with moderate accuracy using network-based models of the underlying connectome.^85,88–92^ The network spread of brain pathology could also explain the propensity of these pathologies to affect hubs, given that the hubs are, by definition, central to the network.^63^

#### Treatment planning

At the time of writing, the only standard clinical use of fMRI is in presurgical planning for the treatment of epilepsy and brain tumours.^93^ These surgeries are often performed on pathological brain tissue surrounding cortical regions crucial for cognition. Accurately mapping and therefore avoiding eloquent tissue is thus an important step in neurosurgical treatment to limit the operation's impact on the patient's quality of life.^49^ Brain mapping can be performed during the operation through direct electrical stimulation to identify regions important for cognitive processes such as language processing or motor planning.^94–96^ However, direct electrical stimulation lengthens the procedure, increasing risk for complications, and cannot be easily used to probe executive functions.^97^

**Figure 3:**
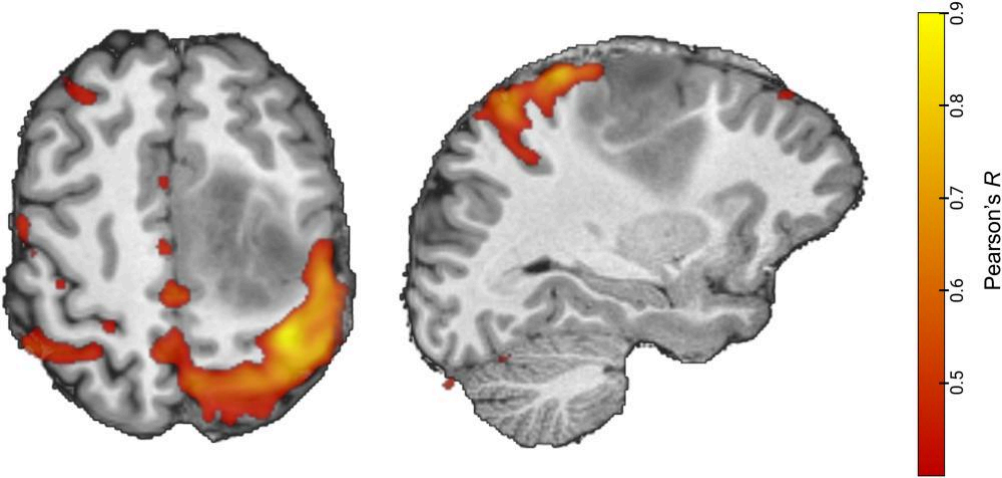
**Illustrative example of resting-state functional connectivity applied to presurgical brain mapping of sensorimotor cortex.** Map of left sensorimotor cortex in patient with a diffuse low-grade glioma derived from seed-based connectivity of a motor cortex voxel.

Task-based fMRI is used in many clinics to map eloquent brain regions before neurosurgical operations.^98^ As the goal of glioma resection is to maximize survival while minimizing cognitive deficit, fMRI is a potentially valuable tool to identify and preserve neural tissue crucial for cognition. However, task-based fMRI can only identify brain networks important for higher-order cognitive functions by employing complex tasks, which are both time-consuming and difficult for patients to perform in the scanner.^29^ Several groups have evaluated the feasibility of resting-state functional imaging for presurgical brain mapping.^7,28,99^ Despite issues surrounding mass effect and neurovascular coupling, these groups have shown that the canonical resting-state networks can be identified in patients with brain tumours, highlighting the feasibility of clinical translation for resting-state network mapping. In Fig. 3, an illustrative example is displayed of resting-state functional connectivity applied to map the sensorimotor cortex in a patient with a low-grade glioma in the supplementary motor areas. Electrocorticography could also play a role in mapping eloquent cortex involved in executive function during glioma resections,^12,100,101^ though its main use in neurosurgery is in guiding the removal of epileptogenic brain tissue.

However, it should also be noted that current functional imaging approaches do not consistently identify eloquent regions and may be less relevant in certain circumstances compared to the connectional anatomy revealed by diffusion tractography.^102,103^ Since gliomas are tumours originating from glial cells, they often surround white matter tracts crucial for healthy brain function, such as the corticospinal tract or arcuate fasciculus. While peritumoural oedema can complicate diffusion tractography around diffuse gliomas, some groups have developed novel correction algorithms to address this problem.^32,104^ Presurgical mapping of white matter pathways using diffusion tractography can be an extremely useful tool in planning glioma resections.

Connectomics has largely been applied to neurosurgery through its potential to preoperatively map eloquent brain tissue. In this way, network neuroimaging helps neurosurgeons navigate the dilemma between maximizing the resection of malignant tissue, to extend patient survival, while minimizing the impact on cortical regions crucial for cognition. While connectomics has clear relevance for the latter goal, an opportunity also exists to apply it to the former, specifically in the case of neurosurgeries to remove glioma tumours.

Gliomas are tumours originating from glial cells and represent the majority of deadly brain cancers. These tumours are difficult to treat because of their capacity to spread along brain networks^105^—therefore, our growing understanding of brain network organization could prove invaluable for developing novel therapeutic strategies to hinder glioma progression. The next sections review historical developments and current concepts in glioma biology, outlining ways in which brain network mapping can advance this clinically important area of research.

## Background in glioma biology

### Historical developments

The term ‘glioma’ was coined in the mid-19th century by the father of modern pathology, Dr Rudolf Virchow, who invoked the new terminology to distinguish between healthy and diseased, neoplastic brain tissue.^106,107^ Virchow's^108^ primary contribution to science was the development of cell theory, which held as a basic tenet that all cells, including diseased cells, are daughters of other cells. Based on this tenet, he was an early proponent of the now-accepted idea that cancers originate from otherwise normal cells, and so he named gliomas for their presumed glial cell origins. Virchow was also the first to classify gliomas, distinguishing between low-grade tumours of reduced cellular density and high-grade tumours with marked contrast to healthy tissue.

Glioma classification became more sophisticated in the beginning of the 20th century with Bailey and Cushing's^109^ seminal 1926 study of over 400 gliomas. Bailey and Cushing were the first to systematically correlate histological and anatomical properties of gliomas with patient outcomes, developing a four-tiered grading system for gliomas that continues to be used in the 21st century.^110^ In their classification, gliomas are classified according to the healthy glial cells they most resemble, leading to glioma subtypes such as ‘astrocytoma’ and ‘oligodendroglioma’. The most lethal and aggressive type of brain tumour was called spongioblastoma multiforme, a term coined to reflect the variegated appearance of cells observed in these tumours, as well as Bailey and Cushing's belief that these tumours had a different cell-of-origin than the healthy glial cells from which gliomas originate.^111^ The term was amended to glioblastoma multiforme (GBM) after the common cellular origin of astrocytomas and GBMs was established.^112^

Harvey Cushing was a disciple of William Halsted^113^, a world-renowned surgeon who popularized the use of radically extensive surgeries to eliminate cancer. Unfortunately, radical surgical strategies were later proven to be of little therapeutic value for most cancers and instead decreased quality of life for cancer patients.^114^ Radical surgeries are of limited potential because of metastasis. Malignant tumours can spread to different organ systems, rendering futile the extensive targeting of the cancer-originating organ, if the cancer is not caught early.^115^ Although primary brain tumours rarely metastasize outside of the brain, adult gliomas are nevertheless diffuse and resistant to surgical treatment.^105^ Even radical hemispherectomies fail to totally eliminate glioma cells, as the cancer can spread along commissural fibres to infiltrate the opposite hemisphere of the brain.^116^ Following the development of surgical techniques and imaging adjuncts to help minimize morbidity, extensive data now support first-line maximal resection whenever possible and subsequent chemotherapy.^117^ Nevertheless, these approaches are not curative for diffuse gliomas because of their infiltration throughout the brain. The clear clinical relevance of glioma cell migration therefore motivated researchers like the Belgian neuropathologist Hans Scherer to study this phenomenon, which had been rather neglected until the 1930s.

In contrast to Bailey and Cushing's classifications, which largely focused on the cytological characteristics of gliomas, Scherer^118^ sought to understand the developmental rules that guide the extension of glial neoplasms.^1^ From observations of the interactions between gliomas and their neural microenvironment, he introduced a distinction between ‘primary structures’—the parts of the glioma that manifest independently of surrounding brain tissue—and ‘secondary structures’, which depend on pre-existing tissue elements for their growth and survival. Secondary structures of gliomas, which are now called Scherer structures,^119^ can be classified into four categories based on the pre-existing tissue elements they rely upon: neurons, the subarachnoid space, blood vessels and white matter. Scherer also argued for an alternative classification system for gliomas, organizing these tumours based on their developmental patterns instead of their cytology, which he presciently recognized to be quite intratumourally variable.

Despite Scherer's concerns about histological characterizations of glioma, the Bailey and Cushing classification remained the primary classification system until the updated World Health Organization guidelines in 2016. The recent World Health Organization 2021 guidelines place emphasis on genomic alterations which have proven to be more prognostic and predictive of therapeutic response than tumour grade.^110,120^ Particularly, prognostic genetic alterations include the isocitrate dehydrogenase (IDH) mutation, which is associated with lower-grade tumours and improved survival, co-deletion of the 1p19q chromosomal arms, which is pathognomonic of oligodendroglioma, and MGMT promoter methylation, which predicts the therapeutic efficacy of temozolomide.^120^ 1p19q co-deletion also predicts responsiveness to radiotherapy as oligodendrogliomas are more radiosensitive than astrocytomas.^105^

Advances in molecular genetic technologies have spurred three main directions of glioma research targeted at better understanding the cancer's cellular heterogeneity, cell-of-origin and relationship with the neural microenvironment. In the next sections, we review the main questions and findings from these areas of research.

### Current research directions

Before outlining current research in neuro-oncology, we will briefly discuss the epidemiology of glioma to help convey why this condition has attracted substantial attention from biomedical researchers and funding bodies. Approximately 100 000 individuals are diagnosed with diffuse glioma each year.^120,121^ While this prevalence is much lower than many other neurological conditions, such as Alzheimer's disease, the mortality rate of diffuse glioma is very high. Glioblastoma, the most lethal glioma subtype which accounts for over 70% of diagnoses, has a median overall survival of 15–17 months.^122^ While median survival of glioblastomas has improved since the current standard of care of temozolomide and radiation was established, these improvements have been modest, as today's patients are expected to live only a couple months longer than the GBM patients from decades ago.^123^ Lower-grade gliomas have a much more favourable prognosis, but they tend to affect younger patients and can convert to glioblastoma; thus, these tumours are also regarded as a research priority.^124^ Overall, the poor survival rate and lack of therapeutic breakthroughs in the clinical management of diffuse gliomas has motivated the application of a litany of cutting-edge genomic and cellular technologies to better understand this disorder, as will be discussed in the next sections.

#### Heterogeneity

In addition to the development of clinically useful genomic markers, the last decade of research has borne witness to improved RNA sequencing technologies, which have characterized the vast heterogeneity of malignant cancers. This heterogeneity exists not just between individuals with the same cancer type, but even within an individual's tumour, as was recognized by Hans Scherer to be the case for glioblastomas. Tumour heterogeneity poses a major challenge to therapeutic development, contributing to the limited efficacy of most pharmacological treatments for primary brain tumours.

**Figure 4:**
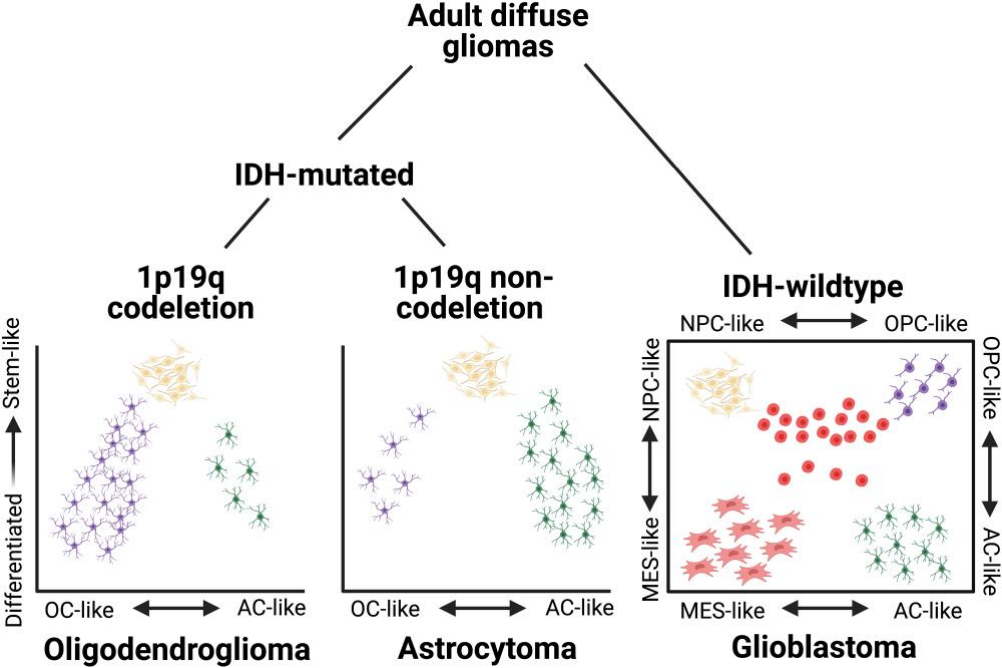
**Inter- and intratumoural heterogeneity of adult diffuse gliomas.** Diffuse low-grade gliomas (i.e. oligodendrogliomas and astrocytomas) exist in a hierarchy with stem-like cells responsible for most mitotic activity, as well as more differentiated subpopulations of oligodendrocyte-like and astrocyte-like cells. GBM cells can transition between four distinct cell states and also contain an intermediate stem-like cell phenotype (shown as the round cells in the graph's centre). OC, oligodendrocyte; AC, astrocyte; MES, mesenchymal; NPC, neural progenitor cell; OPC, oligodendrocyte precursor cell. Created with Biorender.com.

Most of the progress in unpacking intertumoural glioma heterogeneity has been in the identification of genetic distinctions between low-grade glioma and glioblastoma.^120^ The most important prognostic alteration is the IDH mutation, which predicts a less aggressive tumour and is a diagnostic criterion for diffuse low-grade gliomas.^110,125^ When it occurs, the IDH mutation is an early oncogenic event which results in a hypermethylated phenotype.^126^ Among IDH-mutated, low-grade gliomas, the next important alteration is the co-deletion of the 1p19q chromosomal arms. IDH-mutated gliomas that feature this co-deletion possess the morphological characteristics that define oligodendrogliomas, whereas those that lack the co-deletion are classified as astrocytomas (Fig. 4).^127^

IDH-mutated gliomas typically consist of a dominant cell type that is either astrocyte-like or oligodendrocyte-like, yet the tumours will commonly contain a substantial portion of both cell types.^128,129^ In addition to these differentiated cell types, IDH-mutated tumours also host a population of stem-like cells which are the predominant, mitotic cell population of the cancer.^128^ The finding of a brain tumour stem cell (BTSC) population in glioma is strongly supportive of the cancer stem cell hypothesis, which maintains that a relatively small subset of stem-like cells in a given tumour is the main driver of cancer cell proliferation.^129–131^ The BTSCs have emerged as a popular treatment target, given their putative role in driving glioma progression.

Robust intertumoural classification of IDH-wildtype glioblastomas has proven much more challenging. In the early 2010s, many attempts were made to classify GBMs by their bulk transcriptomic features, which eventually settled on three subtypes—proneural, mesenchymal and classical.^132,133^ However, it was soon recognized that bulk RNA sequencing results generally recapitulate the transcriptomic profile of the most dominant cell type in a given sample, thus obscuring the diversity of cell types existing in the tumour.^134,135^ Single-cell RNA sequencing studies have since discovered that each of the canonical transcriptional subtypes can be observed in the same GBM tumour.^134^

Currently, the consensus view is that glioblastoma cells can reside in any of four cell states—astrocyte-like (AC-like), neural progenitor cell-like (NPC-like), oligodendrocyte precursor cell (OPC)-like and mesenchymal—with some flexibility to transition between states (Fig. 4).^130,136^ The largest axis of variation within these cell types can be viewed as a spectrum between a neurodevelopmental state (which includes the AC-like, NPC-like and OPC-like cells) and an immunological state (which includes the mesenchymal cells).^130,136^ This cellular heterogeneity contributes to the resistance of GBM tumours to treatments targeted at any single cell state. Responding to this research, new therapeutic strategies in development seek to convert GBM cells to a state that is more responsive to treatment (such as an astrocyte-like state) before delivering therapy.^130^

Finally, BTSCs have been identified in GBMs as well, although they are less of a distinct cell population within these highly malignant tumours.^137,138^ Multiple GBM cell types, including the mesenchymal state, can initiate tumours when transplanted into a model organism.^118^ Interestingly, even if an isolated cell type is xenografted into a different organism, the resulting tumour displays a heterogeneous distribution of cell states, implying that each GBM cell type is capable of transforming into any other GBM cell type.^136^ Therefore, the original cancer stem cell hypothesis does not perfectly describe GBM, since each GBM cell appears capable of acting like a stem cell.^130^ Nevertheless, the tremendous plasticity of GBM cells has implications for theories regarding the cell-of-origin of the cancer, given that the tumour-initiating cell should, in health, share many characteristics of the resulting tumour.

#### Cell-of-origin

Given the vast cellular heterogeneity of diffuse gliomas, it is unsurprising that the search for the cell-of-origin for this cancer has proven exceedingly difficult. Bailey and Cushing^109^ proposed that gliomas originate from mature astrocytes and oligodendrocytes, thereby deriving the names ‘astrocytoma’ and ‘oligodendroglioma’ in reference to the putative cells-of-origin for these tumours.^106^ However, the neuroscience of their day had yet to acknowledge the existence of stem and progenitor cells in the adult human brain. Currently, it is accepted that at least two types of stem-like cells exist in the adult mammalian brain: neural stem cells (NSCs) and OPCs.^139,140^ Both of these cell types have been proposed as possible cells-of-origin for glioma—the evidence supporting each possibility will be now reviewed.

NSCs are defined by their potential to differentiate into any neural cell, including neurons, astrocytes and oligodendrocytes.^140^ In development, NSCs (more specifically, radial glial cells) both differentiate into neurons and glia and form the scaffold to allow newborn neurons to migrate from the ventricular zone to populate the six cortical layers.^141^ While traditionally, it was thought that neurogenesis ends prenatally, it is now accepted that NSCs continue to divide throughout the lifespan in the dentate gyrus of the hippocampus and the subventricular zone (SVZ).^142^ Adult neurogenesis in the hippocampus has been extensively characterized given its important role in learning and memory.^143^ However, the SVZ represents the largest proliferative niche in the adult mammalian brain,^140^ and several studies have linked gliomagenesis to this location.

In general, studies investigating the anatomical location of gliomagenesis do so by observing the frequency of tumour formation after oncogenic mutations are introduced to select brain locations in model organisms. The earliest experiments invoking this approach introduced carcinogens into the SVZ versus the peripheral cortex of rodents revealing that gliomas form much more frequently when the SVZ is targeted.^144^ Subsequent studies demonstrated that intraventricular injections of avian sarcoma virus could induce neoplasms which migrate out to the surrounding white matter, supporting the idea that gliomas observed outside of the SVZ may nevertheless have originated from cells within this neurogenic niche.^145,146^ This result has been further validated by more recent studies using retroviral injections to introduce combinations of oncogenic mutations to restricted brain areas, finding that malignant transformation is possible within the proliferative niche but exceedingly difficult outside of this area.^146–148^

OPCs are a recently discovered neural cell type which play a major role in regulating myelin plasticity in response to motor and cognitive experiences.^149,150^ Comprising the largest population of dividing cells in the adult mammalian brain,^151,152^ OPCs are a natural candidate as a cell-of-origin for glioma, with several lines of experimental evidence supporting this idea. First, human glioma samples often express histological and genetic markers of OPCs.^153–155^ While the cellular landscape of gliomas is vastly heterogeneous,^129^ glioma cells characterized by OPC markers have been shown to be synaptogenic, proliferative and plastic, supporting the notion that these cells could drive glioma development.^156^ Second, *in vitro* and *in vivo* experiments have demonstrated that OPCs are capable of malignant transformation using the same combinations of mutations which induce gliomas in NSCs.^157–159^ Finally, using an innovative transgenic mouse model, Zong and colleagues^160^ found that even when the initial oncogenic mutations occur in NSCs, aberrant cell growth leading to malignancy is first observed in the cells that have differentiated into OPCs.

Animal models have been useful in elucidating the likely molecular mechanisms underlying early oncogenic events in primary brain cancers. However, it is worth noting that neural proliferative niches are organized differently in humans compared to mice, which poses a limitation for transgenic mouse models to identify the origins of human glioma. In the SVZ of adult mice, NSCs align within a rostral migratory stream directly adjacent to the ependymal zone, and these cells continuously populate the olfactory bulb.^161,162^ The SVZ of adult humans, on the other hand, is composed of four distinct layers: (i) Layer I, composed of ependymal cells that line the lateral ventricles; (ii) Layer II, a hypocellular gap; (iii) Layer III, an astrocytic ribbon composed of NSCs; and (iv) Layer IV, a transitional zone into the brain parenchyma which includes some myelinated axons.^163,164^ Unlike in rodents, NSCs in the adult human astrocytic ribbon do not partake in a chain migration toward the olfactory bulb, as migration from the neurogenic niche declines massively following infancy.^165^

Given the cytoarchitectural and functional differences between the human and rodent SVZ, studies that examine *ex vivo* human tissue, either from neurosurgery cases or post-mortem, can be especially informative. Such studies have confirmed that adult SVZ tissue is indeed proliferative and plastic, capable of forming neurospheres in culture and differentiating into astrocytes, neurons and oligodendrocytes.^163^ Studies of resected human glioma samples have revealed the existence of stem cells in brain tumours as well.^166–168^ These BTSCs are thought to be the drivers of glioma malignancy, given that transplantation of these cells into a living organism can induce a glioma on its own.^169,170^ BTSCs share many characteristics with NSCs, including multipotency, self-renewal and migratory capabilities—a finding which supports the hypothesis that gliomas originate from NSCs.^171^

**Figure 5:**
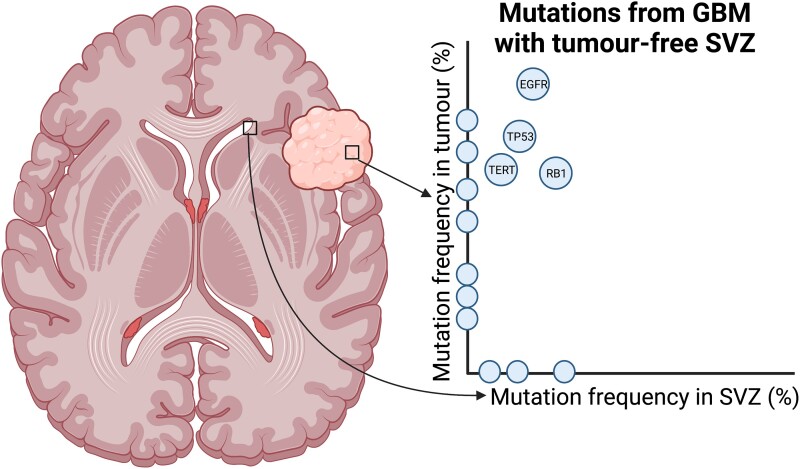
**Schematic of the findings from Lee and colleagues^172^ of genomic evidence for the subventricular origins of glioblastoma.** Biopsies were taken from the healthy-appearing SVZ and the tumour and sequenced (left). In the plot on the right, each datapoint represents a mutated gene, with points located on the *x*- and *y*-axes representing mutations exclusive to the SVZ and tumour, respectively. Datapoints located off of the axes reflect genes that were mutated in both the SVZ and the tumour for this exemplar patient. GBM, glioblastoma; SVZ, subventricular zone. Created with Biorender.com.

Finally, a recent study provided genomic evidence implicating the SVZ as the anatomic origin of adult glioblastomas.^172^ While gliomas commonly contact the lateral ventricles (a radiological finding which relates to poorer survival outcomes),^173,174^ it had been unclear whether glioblastomas that do not appear to involve the SVZ nevertheless harbour tumour-originating cells in that region. Lee and colleagues^172^ obtained samples from the tumour mass and tumour-free SVZ tissue in 14 GBM patients, and in 9 of their subjects, they observed oncogenic mutations in the visually healthy SVZ that were apparent at higher levels within the tumour (Fig. 5). Leveraging genomic analysis of the tumour and SVZ cells, they were able to conclude that the SVZ cells were likely the ancestral population giving rise to the tumour, as opposed to the shared mutations being a result of tumour micro-invasion of the SVZ. It is worth noting that Lee and colleagues did not find shared mutations between healthy SVZ and tumour for the three IDH-mutated gliomas they examined. While differences in cell-of-origin between IDH-mutated and IDH-wildtype gliomas have been proposed,^175^ it is difficult to rule out SVZ origins for low-grade gliomas on the basis of this study alone because of the small IDH-mutated sample size. In summary, the aforementioned human studies support a model where gliomas originate from NSCs in the SVZ, which by malignant transformation become BTSCs that migrate to populate distant cortical locations.

#### Glioma–neuronal interactions

Given the important role of glioma migration in the development and recurrence of primary brain tumours, what do we know about the molecular mechanisms that underlie this behaviour? Building on Scherer's pathology reports, glioma cell migration has now been characterized *in vivo* and has been confirmed to follow pre-existing brain structures, including blood vessels, white matter tracts and brain parenchyma.^105^ Interestingly, migrating glioma cells travel along many of the same routes frequented by immature neurons and stem cells in healthy brain development.^171^ The molecular triggers of cell proliferation and migration are also shared in both normal development and gliomagenesis. In recent years, special attention has been paid to activity-dependent glial cell proliferation, which mediates both healthy brain plasticity as well as malignant glioma progression.

Throughout healthy neurodevelopment, brain circuits are constantly being structurally remodelled and refined through several mechanisms, including synaptic plasticity and myelination. Activity-dependent synaptic plasticity (i.e. long-term potentiation/depression) has been thoroughly characterized as a biological mechanism of learning and memory.^176^ More recently, activity-dependent myelin plasticity has been investigated as another important mediator for behavioural learning.^150^ Through *in viv*o optogenetic stimulation of the premotor cortex, Gibson and colleagues^177^ demonstrated that neuronal activity can elicit the proliferation of OPCs, which differentiate into oligodendrocytes that myelinate the firing neurons. Follow-up studies showed that interfering with activity-dependent myelin plasticity can impair motor skill learning, attention and memory consolidation in mice,^178–180^ implying an important role for this mechanism in neuro-cognitive development.

Myelin plasticity appears to be a double-edged sword, with an integral role in disease as well. Aberrant myelin plasticity has been implicated as a contributor to several neurological conditions, including chemotherapy-induced cognitive impairments,^179,181^ epilepsy,^182^ and, most notably for this review, glioma.^183^ Adopting a similar optogenetic model mentioned previously,^177^ Venkatesh and colleagues^184^ demonstrated that neuronal activity in the premotor cortex can also stimulate glioma cell proliferation and growth. Strikingly, gliomas fail to progress in the absence of growth factors (most crucially, neuroligin-3) secreted by neurons and OPCs during neuronal activity.^183^ Gliomas are dependent on growth factors like neuroligin-3 because they regulate the formation of functional synapses between neurons and glioma cells.^156,183,185^ Interestingly, depolarization of the glioma cells themselves by optogenetics can trigger their proliferation, and conversely, blocking glutamatergic neurotransmission significantly diminishes glioma cell division.^156,183^ Recent work has elucidated in further detail how glioma cell invasion resembles features of normal neural development, and how neuronal activity increases the speed of invasion.^186^

Given that most of the described experiments were performed using animal models, it is natural to ask next whether these principles also apply in the human condition. Applying electrode grids to the tumours of three glioblastoma patients, Venkatesh and colleagues^156^ reported elevated high gamma power in tumour-infiltrated compared to tumour-free tissue, confirming that gliomas are electrically active. A currently unpublished study of 14 GBM patients presented evidence of glioma tumours integrating into cognitive circuits responsive to a lexical retrieval task.^6^ Krishna and colleagues^6^ collected intracranial brain recordings of tumour-infiltrated tissue and the inferior frontal gyrus and found task-induced changes beyond canonical language regions into the tumour, suggesting that the glioma had integrated into the language circuit. This functional integration related to worse language performance and poorer overall survival. In summary, the above studies suggest that neuronal networks contribute to the pathogenesis of diffuse gliomas, though further research is needed to establish their causal role in tumour development in patients.

## Recent research at the intersection of brain mapping and glioma biology

While the fields of brain mapping and glioma biology each have a decades-long history, research that has synthesized both fields to examine the pathophysiology of glioma has emerged relatively recently. Because neuroimaging and tissue biopsies are both routinely acquired during standard clinical care for patients with glioma, there is tremendous opportunity to triangulate mechanisms of glioma pathogenesis by leveraging tools from brain mapping and molecular biology. For example, using retrospective brain imaging data and molecular genetic subtyping, it has been shown that IDH-mutated gliomas predominantly appear in the frontal lobe.^187^ Some investigators have proposed that molecular properties specific to this region, like high glutamatergic flux, may support IDH-mediated gliomagenesis.^188^ More direct testing of the relationship between gliomas and human brain networks, however, requires multimodal interrogation of the complex interactions between the glioma microenvironment and surrounding neural circuitry.

Several prospective research studies have examined how brain tumours affect functional organization, finding that the presence of a tumour disrupts many graph theoretical properties of brain networks.^16,77,189–191^ Other work has proposed mechanisms for how tumour infiltration interferes with cognitive processing, demonstrating that gliomas can alter the information content of electrical brain activity.^192^ However, the relationship between brain networks and brain tumours is bidirectional—tumours have an impact on both local and global brain circuits, while in turn, brain networks (both structural and functional) influence the development and spread of diffuse gliomas.

The latter half of this cycle was explored in a few recent studies utilizing a diversity of methods including rs-fMRI, diffusion tractography, transcriptomics and MEG. Mandal and colleagues^193^ asked whether the non-random spatial distribution of diffuse gliomas could be explained by various biological factors hypothesized to relate to gliomagenesis, including functional hubness, frequency of stem-like cells and spatial transcription patterns of genetic drivers of glioma. Consistent with a prior research finding that neurological disorders tend to affect brain hubs,^81^ a robust correspondence was observed between glioma frequency and functional hubness. In particular, glioma frequency was higher in association cortex compared to primary cortex and was most correlated with graph theoretical measures of connector hubs, brain regions that link together multiple subsystems.

**Figure 6:**
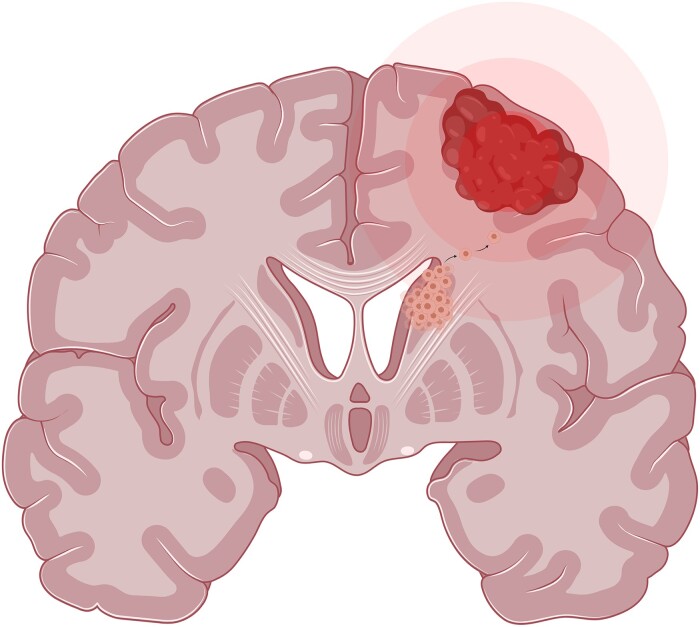
**Proposed model of glioma initiation and development.** This model maintains that adult diffuse gliomas originate in the subventricular zone and then follow intrinsic connectivity pathways to arrive at distant cortical locations. The heterogeneity of glioma locations is explained by tumour origins from different horns of the lateral ventricles. Created with Biorender.com.

The authors also reported a correspondence between glioma frequency and the locations of neurogenic niches and transcriptional patterns of genes involved in synaptic signalling, chromatin reorganization and gliomagenesis. A follow-up study determined that the spatial patterning of diffuse gliomas depends on tumour grade, with more malignant gliomas appearing in regions characterized by normative expression of GBM-related genes and higher connector hubness.^194^ In line with these results, another study found that malignant gliomas preferentially occur in regions with high intrinsic electrical activity as assessed by MEG.^195^ Finally, Mandal and colleagues^196^ identified intrinsic structural and functional networks that coincide with patterns of glioma localization, specifically implicating brain networks connecting neurogenic niches in the SVZ with the cortex. Taken together, these studies are consistent with a model wherein periventricular brain connectivity guides glioma development from the SVZ into distributed cortical locations (Fig. 6).

These findings also suggest that non-invasive measures of brain networks could hold prognostic information regarding the spread and malignancy of glioma tumours. Indeed, some studies have found that measures of functional connectivity are predictive of clinical outcomes for glioma patients, independent of other known prognostic factors.^8,9,197^ A related MEG study found that elevated oscillatory activity within diffuse gliomas was associated with poorer progression free survival outcomes, even after adjusting for potential confounding variables.^10^ The molecular basis for these prognostic imaging markers is currently incompletely understood. One study found that neuroligin-3 expression within resected glioma tissue related to preoperative global oscillatory brain activity,^10^ while another (currently unpublished) MEG study found an association between functional connected tumour regions and synaptogenic factors like thrombospondin-3.^6^

Gaining a better understanding of the molecular factors guiding tumour progression along specific brain networks will be critical for the design of personalized therapies for glioma. For example, clinical trials are currently being run to determine if personalized radiation therapies guided by predictive modelling of recurrence can improve clinical outcomes in glioblastoma (ClinicalTrials.gov Identifier: NCT03477513). An understanding of where a glioma will migrate next can inform pre-emptive strategies to target brain areas that may appear healthy on conventional imaging, but nevertheless harbour malignant cells. Indeed, Wei and colleagues^11^ recently demonstrated that diffusion MRI can detect subtle regional disturbances in the structural connectome that are suggestive of distant glioma invasion and predictive of poor survival outcomes, yet invisible on conventional MRI. Connectomics could develop into a crucial tool to predict and ultimately improve prognosis for patients with diffuse gliomas.

## Conclusion

A better understanding of the neural pathways by which gliomas migrate within the brain could prove critical in the development of novel therapeutic strategies for patients with diffuse glioma. Network neuroscience is poised to tackle this unmet need in the study of glioma pathophysiology by providing non-invasive, *in vivo* access to brain network function in patients. The current research in this area suggests that the analysis of such networks holds prognostic value and describes the common locations of gliomas. Future work should further develop the synthesis between network neuroscience and neuro-oncology, focusing on better defining molecular targets to curtail glioma progression.

## Data Availability

Data sharing is not applicable to this article as no new data were created or analysed in this study.

## Abbreviations

BOLD =: blood–oxygen level dependent
BTSC =: brain tumour stem cell
DMN =: default mode network
GBM =: glioblastoma multiforme
ICA =: independent component analysis
IDH =: isocitrate dehydrogenase
MEG =: magnetoencephalography
NSC =: neural stem cell
OPC =: oligodendrocyte precursor cell
rs-fMRI =: resting-state functional MRI
SVZ =: subventricular zone
